# Quality of life in patients with neurofibromatosis type 1: a nationwide database study in Japan from 2015 to 2019

**DOI:** 10.1265/ehpm.23-00221

**Published:** 2023-12-07

**Authors:** Takashi Yamauchi, Machi Suka

**Affiliations:** Department of Public Health and Environmental Medicine, The Jikei University School of Medicine, 3-25-8 Nishi-shimbashi, Minato-ku, Tokyo 105-8461, Japan

**Keywords:** Intractable and rare disease, Neurofibromatosis type 1, Quality of life, Disease severity, Medical certificate, Nationwide database

## Abstract

**Background:**

This study examined the association between dermatological, neurological, and bone manifestations of neurofibromatosis type 1 (NF1) and quality of life (QoL) in patients with NF1 using a nationwide database of all patients who newly claimed for medical expense subsidies in Japan from 2015 to 2019.

**Methods:**

The Japanese Ministry of Health, Labour and Welfare provided the “National Database of Designated Intractable Diseases of Japan” containing clinical and personal records (“Medical Certificates of Designated Intractable Diseases”) of all patients with NF1 following approval of the study protocol. To examine the association between the severity of symptoms and QoL, multinominal logistic regression analyses were performed, adjusted for potential confounders.

**Results:**

The final study population consisted of 1,487 patients (775 females and 712 males; mean (standard deviation) age, 45.4 (17.9) years). More than 50% and nearly 45% of participants were recorded as having moderate or severe “pain/discomfort” and “anxiety/depression,” respectively. The severity of neurological symptoms was significantly associated with all components of QoL, whereas the severity of dermatological symptoms was significantly associated with only moderate or severe subjective and mental health-related components of QoL, and the severity of bone lesions was associated with only moderate or severe physical health-related components of QoL. Subjective and mental health-related components of QoL tended to be deteriorated more than physical health-related components of QoL in younger and female patients.

**Conclusions:**

Severities of neurological and dermatological symptoms were significantly associated with subjective and mental health-related components of QoL, while the severity of bone symptoms was associated with only moderate and severe deterioration of physical health-related components of QoL.

**Supplementary information:**

The online version contains supplementary material available at https://doi.org/10.1265/ehpm.23-00221.

## Background

Neurofibromatosis type 1 (NF1) is a rare disease that affects approximately one in 2,500 to one in 3,000 people worldwide, regardless of sex or ethnic background [1, 2]. Many manifestations of NF1 affect the skin, the nervous system, and bones [3–5].

Recently, several systematic reviews on the quality of life (QoL) of patients with rare genetic skin diseases, including NF1 [6–10], reported the impact of these diseases and cutaneous/subcutaneous tumor treatment on QoL and physical/psychological functioning. However, the studies included in these reviews had small sample sizes and were conducted only in North American or European countries. In Asian countries, one study in Japan has investigated QoL among patients with a rare genetic skin disease (i.e., NF1) [11], but that study too had a small sample size of 73 adult patients with NF1 from two medical institutes.

Japan has implemented globally unprecedented and comprehensive measures against intractable and rare diseases (*nanbyou* in Japanese) [12, 13]. In January 2015, the Intractable Rare Disease Act (IRDA) was enacted in Japan to socially and financially support patients suffering from these diseases. Meanwhile, patients who meet certain severity grades have been eligible for medical expense subsidies even before 2015, and diagnostic criteria and a disease severity classification for NF1 have been established to standardize the criteria for medical expense subsidies. To receive medical expense subsidies, patients with rare diseases, including NF1, must submit a claim to the prefectural government, along with a medical certificate (“Medical Certificate of Designated Intractable Diseases”) filled in by a physician. The Japanese Ministry of Health, Labour and Welfare (MHLW) has accumulated this information via prefectural governments to create a database of patient medical certificates.

Using this database, we previously examined disease progression in patients with NF1 [14] as well as the loss of social independence among patients with neurofibromatosis type 2 [15, 16]. However, we could not analyze the levels of QoL in these patients because QoL-related items were not included in medical certificates prior to the enactment of the IRDA (i.e., five items for the assessment of QoL were added to the medical certificate in 2015).

In 2015, the MHLW developed a system in which the nationwide database of patients with rare diseases could be provided to researchers for research purposes following approval of their research protocols. Using this nationwide database (“National Database of Designated Intractable Diseases of Japan”), the present study aimed to examine the association between each of dermatological, neurological, and bone manifestations of NF1 and QoL in patients with NF1. A better understanding of QoL among patients with NF1 based on a national registry may contribute to the promotion of physical and psychological well-being of patients with NF1, including support for daily living, social participation/involvement, and coexistence with the disease.

## Methods

### Study population and data source

The MHLW approved the study protocol and provided the database which included information about all patients with NF1 who newly claimed for medical expense subsidies from 2015 to 2019. All personally identifiable information, such as names and addresses, was removed from the database, and each patient was assigned a unique code (identification [ID] number), which was not associated with any identifiable personal information.

**Fig. 1:**
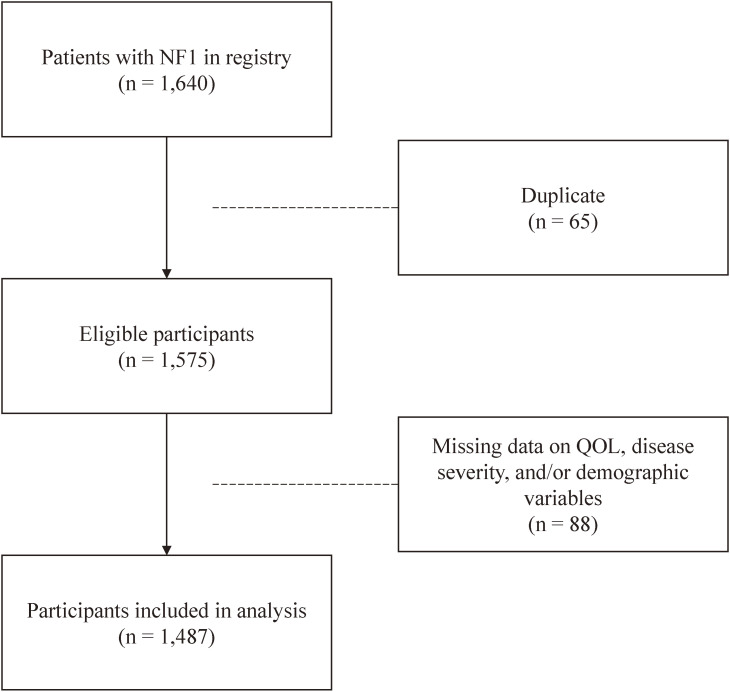
Flowchart of study participant selection

During 2015–2019, the database included data on 1,640 patients who newly (i.e., for the first time) submitted claims to receive medical expense subsidies for NF1 (Fig. 1). Eligibility criteria for study participants were: (1) new registrants during 2015–2019 and (2) no duplicate data. Data duplication was assessed based on ID numbers.

The study protocol was approved by the institutional review board of the Jikei University School of Medicine (No. 33-045(10655)). In addition, following a request from the MHLW, we removed data from Table 3 and Appendix 1 if the number of patients corresponding to the data was less than 10. The results of the present study were obtained from the analyses conducted specifically for research purposes using the National Database of Designated Intractable Diseases of Japan and thus do not correspond to the relevant statistics publicized by the MHLW.

### Variables

The database included the following information: (1) year at registration, (2) severity of dermatological symptoms (D1 to D4), neurological symptoms (N0 to N2), and bone symptoms (B0 to B2), (3) clinical stage of NF1 (Stages 1 to 5), (4) QoL, and (5) demographic variables (i.e., sex and age). Details regarding disease severity and clinical stage classification are provided in Table 1. The severity classification of NF1 in Japan was developed according to criteria set forth by the National Institutes of Health in 1988 [17].

**Table 1:** Participant characteristics (n = 1,487)

	**n**	**(%)**
Year of claim for medical expense subsidies		
2015	261	(18)
2016	267	(18)
2017	295	(20)
2018	359	(24)
2019	305	(21)
Sex		
Male	712	(48)
Female	775	(52)
Age		
0–19 years	121	(8)
20–39 years	474	(32)
40–59 years	538	(36)
>59 years	354	(24)
Family history of NF1		
Present	635	(43)
Absent	597	(40)
Unknown	187	(13)
Dermatological symptoms^a)^		
D1	146	(10)
D2	367	(25)
D3	654	(44)
D4	320	(22)
Neurological symptoms^b)^		
N0	778	(52)
N1	544	(37)
N2	165	(11)
Bone symptoms^c)^		
B0	894	(60)
B1	389	(26)
B2	204	(14)
Clinical stage^d)^		
Stage 1	48	(3)
Stage 2	298	(20)
Stage 3	254	(17)
Stage 4	311	(21)
Stage 5	576	(39)

a) Dermatological symptoms

D1: Pigmented macules and few neurofibromas

D2: Pigmented macules and many neurofibromas

D3: Numerous neurofibromas (>1,000)

D4: Functional impairment or severe physical distress due to plexiform neurofibromas, or malignant peripheral nerve sheath tumor

b) Neurological symptoms

N0: No neurological symptoms

N1: Neurologic symptoms (e.g., paralysis or pain)

N2: Severe/progressive neurological symptoms

c) Bone symptoms

B0: No bone lesions

B1: Mild/moderate bone lesions (e.g., deformity in spine that does not require surgical treatment)

B2: Severe bone lesions

d) Severity classification

Stage 1: D1 and N0 and B0

Stage 2: (D1 or D2) and (N0 or N1) and (B0 or B1)

Stage 3: D3 and N0 and B0

Stage 4: D3 and (N1 or B1)

Stage 5: D4 or N2 or B2

The medical certificate includes five items of QoL (i.e., “mobility,” “self-care,” “usual activities,” “pain/discomfort,” and “anxiety/depression”; Table 2), which were adopted by the MHLW based on the 3-level version of the EQ-5D (EQ-5D-3L) [18]. The EQ-5D is one of the most widely used measures of health-related QoL developed by the EuroQol Group. Regarding the five QoL components, the MHLW requested that these items be used separately (i.e., not combined into a single indicator) when assessing a person’s comprehensive QoL. Accordingly, each of the five QoL components was examined in cross-tabulation and multivariable analyses.

**Table 2:** Quality of life among participants (n = 1,487)

	**n**	**(%)**
Mobility		
M0: I have no problem walking about	1,072	(72)
M1: I have some problems walking about	357	(24)
M2: I am confined to bed	58	(4)
Self-care		
S0: I have no problem with self-care	1,142	(77)
S1: I have some problems washing or dressing myself	267	(18)
S2: I am unable to wash or dress myself	78	(5)
Usual activities (e.g., work, study, housework, family or leisure activities)		
U0: I have no problem performing my usual activities	945	(64)
U1: I have some problems performing my usual activities	471	(32)
U2: I am unable to perform my usual activities	71	(5)
Pain / Discomfort		
PD0: I have no pain or discomfort	647	(44)
PD1: I have moderate pain or discomfort	717	(48)
PD2: I have extreme pain or discomfort	123	(8)
Anxiety / Depression		
AD0: I am not anxious or depressed	841	(57)
AD1: I am moderately anxious or depressed	538	(36)
AD2: I am extremely anxious or depressed	108	(7)

### Statistical analysis

First, to examine the association between the severity of symptoms (i.e., dermatological, neurological, and bone) and QoL, cross-tabulation was performed using Somers’ d values as the indicator of association. Next, multinominal logistic regression analyses were performed with “no problem” for each QoL component (i.e., M0, S0, U0, PD0, and AD0 in Table 2) as the reference category of the dependent variable, adjusted for potential confounders (i.e., age and sex). Since the proportional odds assumption regarding the dependent variable (i.e., QoL) was not met, we did not perform ordinal logistic regression analysis. Adjusted odds ratios (ORs) and 95% confidence intervals (CIs) were calculated.

P < 0.05 was considered statistically significant. All analyses were performed using SPSS version 25 (IBM, Chicago, IL, USA).

## Results

Of the 1,640 newly registered patients during the 5-year study period, 65 were ineligible for inclusion due to duplicate data, and 88 were excluded from analyses due to missing data on disease severity or demographic variables. The final study population consisted of 1,487 participants (775 females and 712 males; mean (SD) age at registration, 45.4 (17.9) years) (Fig. 1).

Table 1 summarizes participant characteristics. Participants with no neurological symptoms (i.e., N0) and no bone symptoms (i.e., B0) accounted for large proportions (52% and 60%, respectively). Nearly 40% of participants were classified as Stage 5.

Proportions of participants who were recorded as having no problem for each QoL component (i.e., M0, S0, U0, PD0, and AD0 in Table 2) ranged from 44% (“pain/discomfort”) to 77% (“self-care”). On the other hand, participants who were recorded as having severe impairment of any QoL component (i.e., M2, S2, U2, PD2, and AD2 in Table 2) accounted for less than 10%. Subjective and mental health-related components of QoL (i.e., “pain/discomfort” and “anxiety/depression”) appeared to be more deteriorated than physical-health related components of QoL (i.e., “mobility,” “self-care,” and “usual activities”) among younger patients (Appendix 1).

Table 3 summarizes the results of cross-tabulation between the severity of dermatological, neurological, and bone symptoms and each QoL component. Severities of neurological and bone symptoms were significantly associated with all components of QoL, whereas the severity of dermatological symptoms was significantly associated with “pain/discomfort” and “anxiety/depression” as well as “mobility,” although the latter component showed a quite small effect size (Somers’ d = 0.05).

**Table 3:** Cross-tabulation between severity of symptoms and quality of life (QoL) (n = 1,487)

**QoL component^a)^**	**Mobility**						**Self-care**						**Usual activities**					
			
	**M0**		**M1**		**M2**		**S0**		**S1**		**S2**		**U0**		**U1**		**U2**	
								
	**n**	**(%)**	**n**	**(%)**	**n**	**(%)**	**n**	**(%)**	**n**	**(%)**	**n**	**(%)**	**n**	**(%)**	**n**	**(%)**	**n**	**(%)**
Dermatological symptoms	Somers’ d = 0.05, P = 0.004						Somers’ d = 0.01, P = 0.56						Somers’ d = 0.03, P = 0.09					
D1	—		—		—		—		—		—		—		—		—	
D2	275	(75)	77	(21)	15	(4)	283	(77)	63	(17)	21	(6)	241	(66)	109	(30)	17	(5)
D3	477	(73)	151	(23)	26	(4)	515	(79)	105	(16)	34	(5)	428	(65)	194	(30)	32	(5)
D4	209	(65)	96	(30)	15	(5)	235	(73)	66	(21)	19	(6)	185	(58)	116	(36)	19	(6)
Neurological symptoms	Somers’ d = 0.26, P < 0.001						Somers’ d = 0.26, P < 0.001						Somers’ d = 0.34, P < 0.001					
N0	655	(84)	109	(14)	14	(2)	684	(88)	74	(10)	20	(3)	617	(79)	144	(19)	17	(2)
N1	350	(64)	181	(33)	13	(2)	398	(73)	131	(24)	15	(3)	294	(54)	231	(42)	19	(3)
N2	67	(41)	67	(41)	31	(19)	60	(36)	62	(38)	43	(26)	34	(21)	96	(58)	35	(21)
Bone symptoms	Somers’ d = 0.13, P < 0.001						Somers’ d = 0.11, P < 0.001						Somers’ d = 0.14, P < 0.001					
B0	690	(77)	174	(19)	30	(3)	728	(81)	124	(14)	42	(5)	620	(69)	237	(27)	37	(4)
B1	268	(69)	105	(27)	16	(4)	287	(74)	79	(20)	23	(6)	227	(58)	142	(37)	20	(5)
B2	114	(56)	78	(38)	12	(6)	127	(62)	64	(31)	13	(6)	98	(48)	92	(45)	14	(7)

**QoL component^a)^**	**Pain / Discomfort**						**Anxiety / Depression**					
		
	**PD0**		**PD1**		**PD2**		**AD0**		**AD1**		**AD2**	
					
	**n**	**(%)**	**n**	**(%)**	**n**	**(%)**	**n**	**(%)**	**n**	**(%)**	**n**	**(%)**
Dermatological symptoms	Somers’ d = 0.13, P < 0.001						Somers’ d = 0.08, P < 0.001					
D1	—		—		—		—		—		—	
D2	179	(49)	168	(46)	20	(5)	225	(61)	123	(34)	19	(5)
D3	270	(41)	338	(52)	46	(7)	353	(54)	252	(39)	49	(7)
D4	110	(34)	161	(50)	49	(15)	167	(52)	120	(38)	33	(10)
Neurological symptoms	Somers’ d = 0.32, P < 0.001						Somers’ d = 0.19, P < 0.001					
N0	465	(60)	284	(37)	29	(4)	507	(65)	238	(31)	33	(4)
N1	134	(25)	350	(64)	60	(11)	271	(50)	226	(42)	47	(9)
N2	48	(29)	83	(50)	34	(21)	63	(38)	74	(45)	28	(17)
Bone symptoms	Somers’ d = 0.09, P < 0.001						Somers’ d = 0.07, P = 0.008					
B0	428	(48)	397	(44)	69	(8)	531	(59)	304	(34)	59	(7)
B1	140	(36)	218	(56)	31	(8)	202	(52)	160	(41)	27	(7)
B2	79	(39)	102	(50)	23	(11)	108	(53)	74	(36)	22	(11)

a) Classification of each component

Mobility

M0: I have no problem walking about

M1: I have some problems walking about

M2: I am confined to bed

Self-care

S0: I have no problem with self-care

S1: I have some problems washing or dressing myself

S2: I am unable to wash or dress myself

Usual activities (e.g., work, study, housework, family or leisure activities)

U0: I have no problem performing my usual activities

U1: I have some problems performing my usual activities

U2: I am unable to perform my usual activities

Pain / Discomfort

PD0: I have no pain or discomfort

PD1: I have moderate pain or discomfort

PD2: I have extreme pain or discomfort

Anxiety / Depression

AD0: I am not anxious or depressed

AD1: I am moderately anxious or depressed

AD2: I am extremely anxious or depressed

Table 4 summarizes the results of multinominal logistic regression analyses with each component of QoL as the dependent variable. For all components of QoL, ORs were significantly higher for those with moderate and severe neurological symptoms than for those with no neurological symptoms. For “mobility” and “usual activities,” ORs were significantly higher for those with moderate and severe bone symptoms than for those with no bone symptoms. On the other hand, for “pain/discomfort” and “anxiety/depression,” ORs were significantly higher for those with higher severity of dermatological symptoms (i.e., D2 to D4) than for those with the lowest severity (i.e., D1). Regarding sex and age, compared with the physical-health related components of QoL, subjective and mental health-related components of QoL tended to be more deteriorated among younger and female patients.

**Table 4:** Multinominal logistic regression analysis using severity of symptoms as the independent variable (n = 1,487)

**QoL component^a),b)^**	**Mobility**				**Self-care**				**Usual activities**			
			
	**M1**		**M2**		**S1**		**S2**		**U1**		**U2**	
					
	**OR^c)^**	**(95% CI)**	**OR^c)^**	**(95% CI)**	**OR^c)^**	**(95% CI)**	**OR^c)^**	**(95% CI)**	**OR^c)^**	**(95% CI)**	**OR^c)^**	**(95% CI)**
Dermatological symptoms (Reference: D1)	1.1	(0.97–1.3)	1.2	(0.8–1.7)	0.9	(0.8–1.1)	1.0	(0.7–1.4)	1.0	(0.9–1.2)	1.1	(0.8–1.6)
Neurological symptoms (Reference: N0)	2.9	(2.4–3.5)	9.1	(5.8–14.3))	3.3	(2.7–4.1)	8.6	(5.7–12.7)	3.6	(3.0–4.4)	10.1	(6.7–15.4)
Bone symptoms (Reference: B0)	1.6	(1.4–1.9)	1.5	(1.04–2.3)	1.7	(1.4–2.0)	1.3	(0.9–1.9)	1.6	(1.3–1.8)	1.5	(1.1–2.2)
Age (Reference: 0–19 years)												
20–39 years	1.5	(0.9–2.6)	1.6	(0.2–14.4)	1.0	(0.5–1.8)	0.5	(0.1–1.9)	0.9	(0.6–1.5)	0.9	(0.2–4.6)
40–59 years	1.6	(0.9–2.8)	4.1	(0.5–33.0)	1.4	(0.8–2.6)	1.4	(0.4–4.7)	1.1	(0.7–1.8)	2.3	(0.5–10.6)
>59 years	4.7	(2.6–8.5)	51.6	(6.6–403.6)	3.3	(1.8–6.1)	12.7	(4.0–40.1)	2.3	(1.4–3.8)	22.5	(4.9–102.6)
Sex (Reference: male)	1.0	(0.8–1.3)	1.1	(0.6–2.0)	1.0	(0.7–1.3)	1.0	(0.6–1.7)	1.0	(0.8–1.3)	0.9	(0.5–1.4)

**QoL component^a),b)^**	**Pain / Discomfort**				**Anxiety / Depression**			
		
	**PD1**		**PD2**		**AD1**		**AD2**	
			
	**OR^c)^**	**(95% CI)**	**OR^c)^**	**(95% CI)**	**OR^c)^**	**(95% CI)**	**OR^c)^**	**(95% CI)**
Dermatological symptoms (Reference: D1)	1.4	(1.2–1.6)	2.0	(1.6–2.6)	1.1	(1.01–1.3)	1.4	(1.1–1.8)
Neurological symptoms (Reference: N0)	2.5	(2.1–3.0)	5.0	(3.7–6.8)	1.7	(1.4–2.0)	2.9	(2.2–3.9)
Bone symptoms (Reference: B0)	1.3	(1.1–1.8)	1.3	(0.96–1.7)	1.1	(0.98–1.3)	1.3	(0.97–1.7)
Age (Reference: 0–19 years)								
20–39 years	2.0	(1.3–3.2)	2.7	(0.97–7.6)	1.9	(1.2–3.0)	1.3	(0.5–3.3)
40–59 years	2.0	(1.3–3.2)	3.3	(1.2–9.0)	2.3	(1.4–3.8)	2.0	(0.8–5.0)
>59 years	1.5	(0.96–2.5)	3.2	(1.1–9.0)	2.1	(1.3–3.5)	3.3	(1.3–8.5)
Sex (Reference: male)	1.4	(1.1–1.8)	1.3	(0.8–1.9)	1.4	(1.1–1.8)	1.7	(1.1–2.6)

a) Classification of each component

Mobility

M0: I have no problem walking about

M1: I have some problems walking about

M2: I am confined to bed

Self-care

S0: I have no problem with self-care

S1: I have some problems washing or dressing myself

S2: I am unable to wash or dress myself

Usual activities (e.g., work, study, housework, family or leisure activities)

U0: I have no problem performing my usual activities

U1: I have some problems performing my usual activities

U2: I am unable to perform my usual activities

Pain / Discomfort

PD0: I have no pain or discomfort

PD1: I have moderate pain or discomfort

PD2: I have extreme pain or discomfort

Anxiety / Depression

AD0: I am not anxious or depressed

AD1: I am moderately anxious or depressed

AD2: I am extremely anxious or depressed

b) Reference: M0, S0, U0, PD0, and AD0

c) OR: odds ratio

Appendices 2 and 3 show the results of cross-tabulation between NF1 clinical stage and QoL and multinominal logistic regression analyses, respectively. For all components of QoL, a higher clinical stage was significantly associated with more deteriorated QoL (Appendix 2). On the other hand, Stage 2 and Stage 3 NF1 showed a similar trend of associations with all QoL components, although the estimation ranges of ORs and their 95% CIs in multinominal logistic regression analyses were poor due to the relatively small number of participants classified as Stage 1 (i.e., the reference category) (Appendix 3).

## Discussion

This study examined the association between dermatological, neurological, and bone manifestations of NF1 and QoL using a nationwide database of all patients with NF1 who newly claimed for medical expense subsidies in Japan from 2015 to 2019. Multinominal logistic regression analyses revealed that (1) the severity of neurological symptoms was significantly associated with all components of QoL, (2) the severity of bone symptoms was significantly associated with physical health-related components of QoL, and (3) the severity of dermatological symptoms was significantly associated with only subjective and mental health-related components of QoL.

During the 5-year study period, 1,575 patients with NF1 newly claimed for medical expense subsidies (Fig. 1). We previously reported that 352 patients submitted claims for subsidies for NF1 in Japan in 2008, and the annual number of patient registration remained ≥300 thereafter [14]. This suggests that the number of patients with NF1 who newly claimed for medical expense subsidies followed a similar trend pre- and post-enactment of IRDA in 2015.

As shown in Table 2, 56% and 43% of participants were recorded as having moderate or severe “pain/discomfort” and “anxiety/depression,” respectively. In addition, the severity of dermatological symptoms was significantly associated with only subjective and mental health-related components of QoL. Furthermore, moderate or severe impairment of physical health-related components of QoL were significant only for those aged 60 years or older, whereas moderate impairment of subjective and mental health-related components of QoL were significant for those aged 20–39 and 40–59 years, relative to those aged 19 years or younger. A systematic review [8] reported that NF1 can affect psychological well-being in patients, with more pronounced negative effects (e.g., depressive symptoms) on women than on men [19]. This may be due to the negative impact of NF1, such as cosmetic disfigurement, negative body image, and appearance-related concerns [20, 21]. Our findings suggest that, regarding the impact of NF1 on the daily and social lives of patients, subjective and mental health-related components of QoL tend to be more deteriorated than physical health-related components of QoL in patients with higher severity of dermatological symptoms, as well as younger and female patients.

In the present study, participants aged 60 years or older had substantially higher ORs for moderately and severely impaired “mobility,” “self-care,” and “usual activities” compared with those aged 19 years or younger (Table 4). Nevertheless, the severity of neurological symptoms was strongly associated with all components of QoL in multinominal logistic regression analyses adjusted for age. Although detailed information about neurological symptoms was not included in the national database, progressive neurological symptoms, especially paralysis and pain, may more strongly deteriorate multiple components of QoL than other symptoms/lesions in patients with NF1. According to a previous systematic review [8], one of the most important characteristics of NF1 is the presence of acute/chronic pain and physical discomfort [22]. Neurological symptoms can disturb sleep and daily functioning and increase fatigue, leading to deterioration of “mobility,” “self-care,” and “usual activities” (e.g., job, studying) in patients with NF1.

The severity of bone symptoms was significantly associated with physical activity-related components of QoL, even after adjusting for age in the multivariable analyses. Previous studies found that NF1 causes movement and mobility impairments due to skeletal disorders and tumors [8, 22]. NF1 also negatively affects patient autonomy; a previous systematic review [8] reported that this deterioration in autonomy can influence, for example, patients’ usual activities, including vocational decision-making and social participation/interaction [23].

Regarding clinical stage, for all components of QoL, a higher clinical stage was significantly associated with more deteriorated QoL (Appendices 2 and 3). However, Stage 2 and Stage 3 NF1 showed a similar trend of associations with QoL, regardless of the component. Although only less than 10% of participants were recorded as having severe impairment of each QoL component at the time of registration in the database (Table 2), future follow-up studies will be needed to examine changes in the association between clinical stage and QoL among patients with NF1.

### Strengths and limitations

The database used in the present study included information about all patients with NF1 who newly submitted medical certificates filled out by physicians to receive medical expense subsidies during the 5-year study period. The large sample size (approximately 1,500 patients with NF1) allowed the authors to accurately analyze the characteristics of QoL among these patients. There are, however, some limitations worth noting. First, patients with NF1 who did not apply for medical expense subsidies during 2015–2019 were not included in the study. For example, individuals with subclinical NF1 who did not require frequent medical care may not have submitted claims for subsidies, and hence, may not have been included in the database analyzed in the present study; this could potentially have resulted in selection bias. Second, while a previous study in Japan revealed that QoL tended to be more deteriorated in patients with NF1 than in healthy volunteers [11], we could not compare the levels of QoL between patients with NF1 and the general population in Japan. In addition, the assessment of QoL for each patient was based on a medical certificate filled in by a physician. Third, as this study was cross-sectional in design, it was not possible to determine robust causality between disease severity, clinical stage, and QoL. Fourth, the database lacked information on comorbidities for each patient, and the possibility cannot be ruled out that QoL might have been affected by underlying disorders other than NF1. Finally, study participants were limited to Japanese patients. Although previous studies have found no significant differences in the incidence/prevalence of NF1 across countries and ethnicities [1, 2], caution should be exercised when generalizing the present findings to different populations.

## Conclusions

In the present epidemiological study, more than 50% and nearly 45% of participants were recorded as having moderate or severe “pain/discomfort” and “anxiety/depression,” respectively. Severities of neurological and dermatological symptoms were significantly associated with subjective and mental health-related components of QoL, while the severity of bone symptoms was associated with only moderate and severe deterioration of physical health-related components of QoL.

## Abbreviations

IRDA: Intractable Rare Disease Act
MHLW: Ministry of Health, Labour and Welfare
NF1: Neurofibromatosis type 1
QoL: Quality of life

## Supplementary information

**Additional file 1:** **Appendix 1.** Distribution of five components of quality of life by age.

**Additional file 2:** **Appendix 2.** Cross-tabulation between clinical stage and quality of life (QoL) (n = 1,487). **Appendix 3.** Multinominal logistic regression analysis using clinical stage as the independent variable (n = 1,487).

## Declarations

### Data availability

Data cannot be shared for privacy or ethical reasons.
