# Supplementary material for: Quality of life in patients with neurofibromatosis type 1: a nationwide database study in Japan from 2015 to 2019
Source: Environ Health Prev Med. 2023 Dec 7;28:77. doi: 10.1265/ehpm.23-00221 (PMC10711372; doi:10.1265/ehpm.23-00221)
Supplement: Supplementary file 1 — Additional file 1: Appendix 1. Distribution of five components of quality of life by age. [file ehpm-28-077-s001.pptx]

## Slide 1
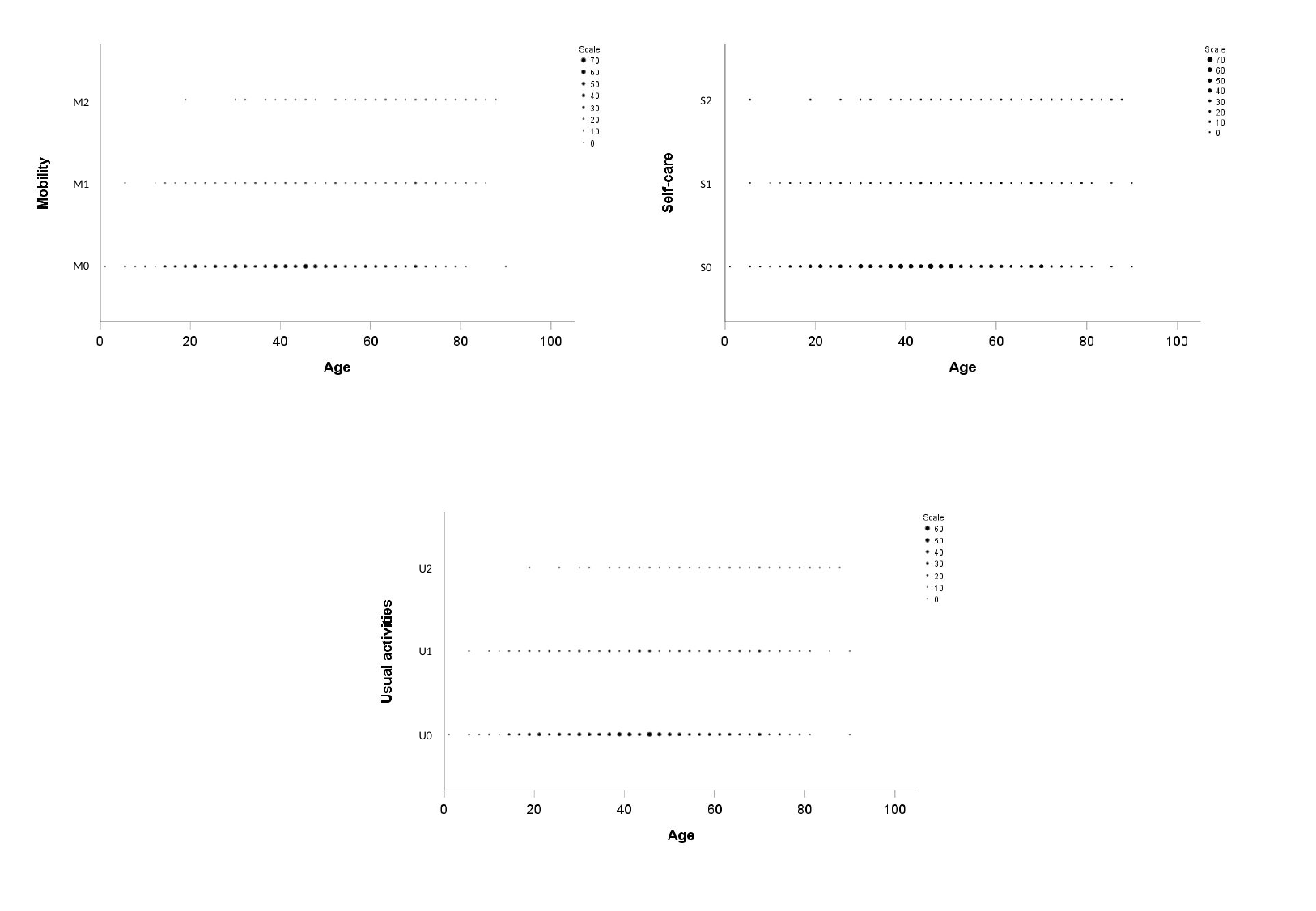

S2
M2
M1
S1
M0
S0
U2
U1
U0

## Slide 2
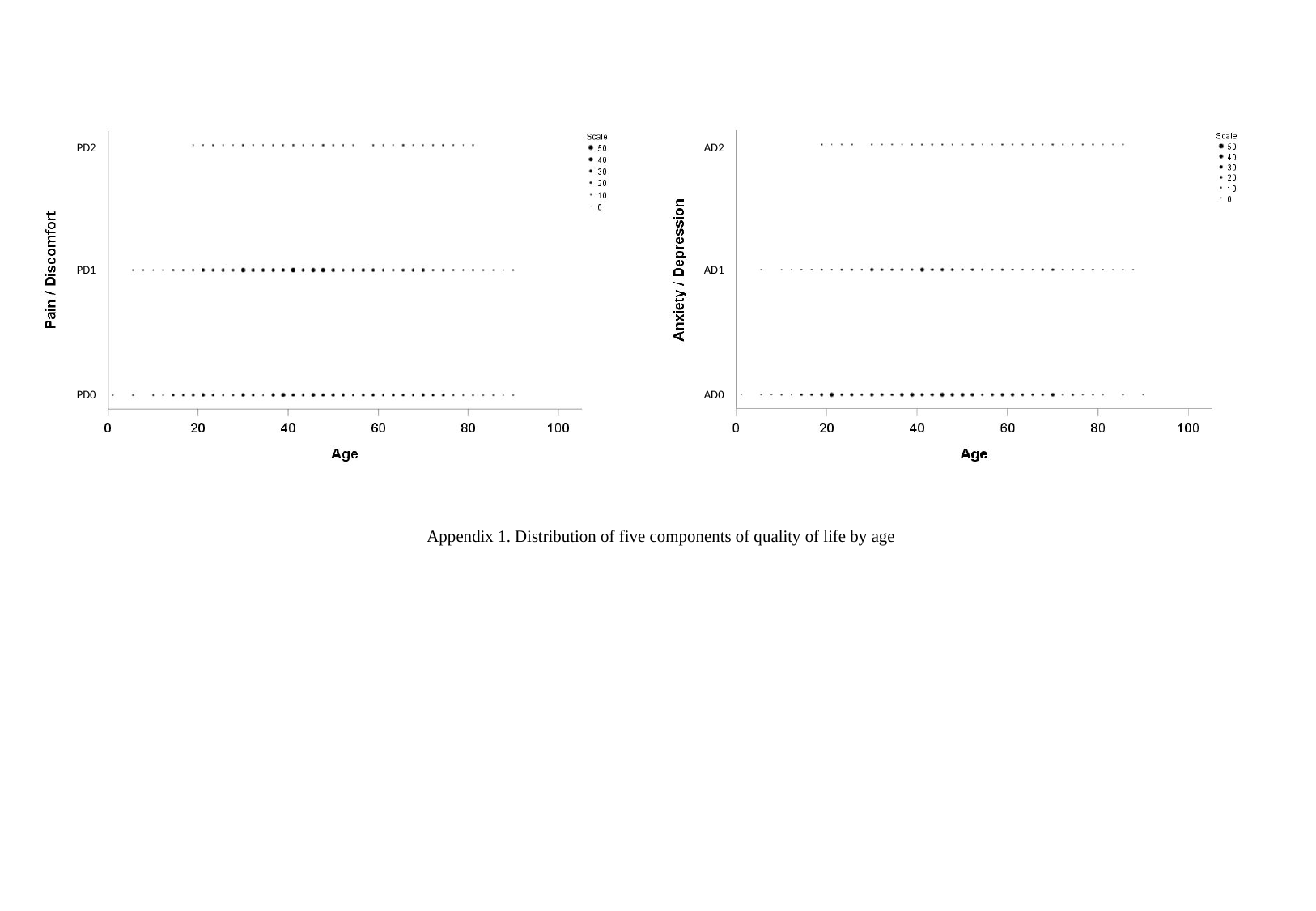

PD2
AD2
PD1
AD1
PD0
AD0
Appendix 1. Distribution of five components of quality of life by age
