# Supplementary material for: Quality of life in patients with neurofibromatosis type 1: a nationwide database study in Japan from 2015 to 2019
Source: Environ Health Prev Med. 2023 Dec 7;28:77. doi: 10.1265/ehpm.23-00221 (PMC10711372; doi:10.1265/ehpm.23-00221)
Supplement: Supplementary file 2 — Additional file 2: Appendix 2. Cross-tabulation between clinical stage and quality of life (QoL) (n = 1,487). Appendix 3. Multinominal logistic regression analysis using clinical stage as the independent variable (n = 1,487). [file ehpm-28-077-s002.docx]

| Appendix 2. Cross-tabulation between clinical stage and quality of life (QoL) (n=1,487) | | | | | | | | | | | | | | | | | |
| --- | --- | --- | --- | --- | --- | --- | --- | --- | --- | --- | --- | --- | --- | --- | --- | --- | --- |
| QoL component ^a)^ | Mobility | | | | |  | Self-care | | | | |  | Usual activities | | | | |
|  | M0 |  | M1 |  | M2 |  | S0 |  | S1 |  | S2 |  | U0 |  | U1 |  | U2 |
|  | n |  | n |  | n |  | n |  | n |  | n |  | n |  | n |  | n |
| Clinical stage | Somers' d=0.19, P<0.001 | | | | |  | Somers' d=0.17, P<0.001 | | | | |  | Somers' d=0.21, P<0.001 | | | | |
| Stage 1 | — |  | — |  | — |  | — |  | — |  | — |  | — |  | — |  | — |
| Stage 2 | 252 |  | — |  | — |  | 262 |  | — |  | — |  | 232 |  | — |  | — |
| Stage 3 | 218 |  | — |  | — |  | 228 |  | — |  | — |  | 208 |  | — |  | — |
| Stage 4 | 220 |  | — |  | — |  | 241 |  | — |  | — |  | 189 |  | — |  | — |
| Stage 5 | 336 |  | 200 |  | 40 |  | 366 |  | 155 |  | 55 |  | 276 |  | 252 |  | 48 |
|  |  |  |  |  |  |  |  |  |  |  |  |  |  |  |  |  |  |
| Appendix 2. Cross-tabulation between clinical stage and quality of life (QoL) (n=1,487) (continued) | | | | | | | | | | | |  |  |  |  |  |  |
| QoL component ^a)^ | Pain / Discomfort | | | | |  | Anxiety / Depression | | | | |  |  | | | | |
|  | PD0 |  | PD1 |  | PD2 |  | AD0 |  | AD1 |  | AD2 |  |  |  |  |  |  |
|  | n |  | n |  | n |  | n |  | n |  | n |  |  |  |  |  |  |
| Clinical stage | Somers' d=0.18, P<0.001 | | | | |  | Somers' d=0.12, P<0.001 | | | | |  |  |  |  |  |  |
| Stage 1 | — |  | — |  | — |  | — |  | — |  | — |  |  |  |  |  |  |
| Stage 2 | 159 |  | 127 |  | 12 |  | 192 |  | 92 |  | 14 |  |  |  |  |  |  |
| Stage 3 | 148 |  | — |  | — |  | 163 |  | 80 |  | 11 |  |  |  |  |  |  |
| Stage 4 | 99 |  | 190 |  | 22 |  | 159 |  | 130 |  | 22 |  |  |  |  |  |  |
| Stage 5 | 203 |  | 293 |  | 80 |  | 288 |  | 227 |  | 61 |  |  |  |  |  |  |
| a) Classification of each component Mobility M0: I have no problem walking about M1: I have some problems walking about M2: I am confined to bed Self-care S0: I have no problem with self-care S1: I have some problems washing or dressing myself S2: I am unable to wash or dress myself Usual activities (e.g., work, study, housework, family or leisure activities) U0: I have no problem performing my usual activities U1: I have some problems performing my usual activities U2: I am unable to perform my usual activities Pain / Discomfort PD0: I have no pain or discomfort PD1: I have moderate pain or discomfort PD2: I have extreme pain or discomfort Anxiety / Depression AD0: I am not anxious or depressed AD1: I am moderately anxious or depressed AD2: I am extremely anxious or depressed | | | | | | | | | | | | | | | | | |

| Appendix 3. Multinominal logistic regression analysis using clinical stage as the independent variable (n=1,487) | | | | | | | | | | | | | | | | | |
| --- | --- | --- | --- | --- | --- | --- | --- | --- | --- | --- | --- | --- | --- | --- | --- | --- | --- |
| QoL component ^a),b)^ | Mobility | | | | |  | Self-care | | | | |  | Usual activities | | | | |
|  | M1 | |  | M2 | |  | S1 | |  | S2 | |  | U1 | |  | U2 | |
|  | OR ^c)^ | (95% CI) |  | OR ^c)^ | (95% CI) |  | OR ^c)^ | (95% CI) |  | OR ^c)^ | (95% CI) |  | OR ^c)^ | (95% CI) |  | OR ^c)^ | (95% CI) |
| Clinical stage (Reference: Stage 1) |  |  |  |  |  |  |  |  |  |  |  |  |  |  |  |  |  |
| Stage 2 | 6.2 | (0.8-46.1) |  | 0.3 | (0.1-2.9) |  | 2.3 | (0.5-10.1) |  | 0.5 | (0.1-4.6) |  | 1.4 | (0.6-3.3) |  | 0.4 | (0.1-3.9) |
| Stage 3 | 4.0 | (0.5-30.7) |  | 0.2 | (0.1-1.8) |  | 1.2 | (0.3-5.3) |  | 0.5 | (0.1-4.7) |  | 0.8 | (0.3-2.1) |  | 0.4 | (0.1-3.3) |
| Stage 4 | 11.4 | (1.5-85.0) |  | 0.4 | (0.1-3.3) |  | 4.2 | (0.97-18.0) |  | 0.5 | (0.1-4.4) |  | 2.9 | (1.2-6.7) |  | 0.5 | (0.1-4.6) |
| Stage 5 | 21.6 | (2.9-158.8) |  | 2.0 | (0.2-16.1) |  | 8.0 | (1.9-33.7) |  | 3.5 | (0.2-27.6) |  | 5.0 | (2.1-11.0) |  | 3.0 | (0.4-24.0) |
| Age (Reference: 0-19 years) |  |  |  |  |  |  |  |  |  |  |  |  |  |  |  |  |  |
| 20-39 years | 1.4 | (0.8-2.4) |  | 1.6 | (0.2-14.4) |  | 0.9 | (0.5-1.6) |  | 0.6 | (0.2-1.9) |  | 0.8 | (0.5-1.3) |  | 1 | (0.2-4.9) |
| 40-59 years | 1.4 | (0.8-2.5) |  | 3.9 | (0.5-30.9) |  | 1.2 | (0.7-2.1) |  | 1.3 | (0.4-3.9) |  | 0.9 | (0.6-1.4) |  | 2.2 | (0.5-10.1) |
| >59 years | 3.6 | (2.1-6.4) |  | 35.4 | (4.6-271.5) |  | 2.3 | (1.3-4.1) |  | 8.0 | (2.7-23.7) |  | 1.4 | (0.9-2.2) |  | 15.8 | (3.6-69.6) |
| Sex (Reference: male) | 1.0 | (0.7-1.2) |  | 0.9 | (0.5-1.7) |  | 1.0 | (0.7-1.2) |  | 0.9 | (0.5-1.4) |  | 1.0 | (0.8-1.2) |  | 0.7 | (0.4-1.2) |
|  |  |  |  |  |  |  |  |  |  |  |  |  |  |  |  |  |  |
| Appendix 3. Multinominal logistic regression analysis using clinical stage as the independent variable (n=1,487) (continued) | | | | | | | | | | | |  |  |  |  |  |  |
| QoL component ^a),b)^ | Pain / Discomfort | | | | |  | Anxiety / Depression | | | | |  |  | | | | |
|  | PD1 | |  | PD2 | |  | AD1 | |  | AD2 | |  |  | |  |  | |
|  | OR ^c)^ | (95% CI) |  | OR ^c)^ | (95% CI) |  | OR ^c)^ | (95% CI) |  | OR ^c)^ | (95% CI) |  |  |  |  |  |  |
| Clinical stage (Reference: Stage 1) |  |  |  |  |  |  |  |  |  |  |  |  |  |  |  |  |  |
| Stage 2 | 2.8 | (1.3-5.8) |  | — |  |  | 1.8 | (0.8-3.8) |  | — |  |  |  |  |  |  |  |
| Stage 3 | 2.4 | (1.1-5.1) |  | — |  |  | 1.7 | (0.8-3.8) |  | — |  |  |  |  |  |  |  |
| Stage 4 | 6.9 | (3.2-14.7) |  | — |  |  | 2.9 | (1.3-6.3) |  | — |  |  |  |  |  |  |  |
| Stage 5 | 5.2 | (2.5-10.7) |  | — |  |  | 2.9 | (1.4-6.2) |  | — |  |  |  |  |  |  |  |
| Age (Reference: 0-19 years) |  |  |  |  |  |  |  |  |  |  |  |  |  |  |  |  |  |
| 20-39 years | 1.9 | (1.2-2.9) |  | 2.6 | (0.97-7.1) |  | 1.8 | (1.1-2.9) |  | 1.2 | (0.5-3.1) |  |  |  |  |  |  |
| 40-59 years | 1.9 | (1.2-2.9) |  | 3.3 | (1.2-8.7) |  | 2.2 | (1.4-3.5) |  | 1.9 | (0.8-4.7) |  |  |  |  |  |  |
| >59 years | 1.3 | (0.8-2.1) |  | 3.1 | (1.1-8.5) |  | 1.9 | (1.2-3.1) |  | 3.0 | (1.2-7.6) |  |  |  |  |  |  |
| Sex (Reference: male) | 1.4 | (1.1-1.7) |  | 1.2 | (0.8-1.7) |  | 1.4 | (1.1-1.7) |  | 1.6 | (1.05-2.4) |  |  |  |  |  |  |
| a) Classification of each component Mobility M0: I have no problem walking about M1: I have some problems walking about M2: I am confined to bed Self-care S0: I have no problem with self-care S1: I have some problems washing or dressing myself S2: I am unable to wash or dress myself Usual activities (e.g., work, study, housework, family or leisure activities) U0: I have no problem performing my usual activities U1: I have some problems performing my usual activities U2: I am unable to perform my usual activities Pain / Discomfort PD0: I have no pain or discomfort PD1: I have moderate pain or discomfort PD2: I have extreme pain or discomfort Anxiety / Depression AD0: I am not anxious or depressed AD1: I am moderately anxious or depressed AD2: I am extremely anxious or depressed b) Reference: M0, S0, U0, PD0, and AD0 c) OR: odds ratio | | | | | | | | | | | | | | | | | |
